# Can only intelligent children do mind reading: The relationship between intelligence and theory of mind in 8 to 11 years old

**DOI:** 10.1186/1744-9081-4-51

**Published:** 2008-11-11

**Authors:** Anto P Rajkumar, Simpson Yovan, Anoop L Raveendran, Paul Swamidhas Sudhakar Russell

**Affiliations:** 1 Child and Adolescent Psychiatry Unit, Department of Psychiatry, Christian Medical College, Vellore, Tamil Nadu – 632002, India

## Abstract

**Background:**

The mind reading ability of children has evoked wide interest, but its relationship with general cognitive abilities remains obscure.

**Methods:**

We studied the relationship between the mind reading ability and general intelligence. Children (N = 105) between 8 to 11 years from educational institutions were assessed for the mind reading ability using *Picture Sequencing Task *and *Unexpected Contents Theory of Mind task*. We used *Strengths and Difficulties Questionnaire *to rule out psychiatric morbidity. An independent investigator quantified intelligence and adaptive behavior with *Binet- Kamat Test of intelligence *and *Vineland Adaptive Behavior Scale *respectively. We employed bivariate and multivariate statistical tests.

**Results:**

We demonstrated that mind reading ability was not significantly related to general intelligence or its domains except for the social intelligence after controlling the confounders methodologically and statistically.

**Conclusion:**

These findings argue that mind reading skill exists as an independent cognitive domain and has clinical, research as well as educational implications.

## Background

Can we explain children's ability to understand human minds by their sheer sense of intelligence? Will anyone of us bravely bet that the most intelligent student in a classroom is the best in that class to read minds? The cognitive ability to impute mental states to the self and others meaningfully, to predict and explain the intention of behaviors in terms of mental states is named as *Mind reading *or *Theory of Mind *(ToM) [1,2]. ToM serves children to understand that others may hold as well as act upon beliefs different from theirs and appreciating such alternative perspectives is essential for successful social interaction.

Research on ToM had usually focused on its origin, development [3] and deficits [4]. Theory of Mind is said to be active even in 15-month toddlers [5] and be deficient in a variety of psychiatric [6-8] as well as neurological disorders [9,10]. However, ToM's independent existence as a specific cognitive domain is still controversial [11,12].

To resolve this debate studying the relationship between ToM and general cognitive abilities is imperative. Previous studies have investigated the relationship between ToM and executive functions [13], language ability [14], and episodic memory [15] among children with compromised intelligence [16] or psychopathology [17,18]. Among the various general cognitive abilities, Intelligence Quotient (IQ) is considered the main confounding factor in studying ToM [19]. Currently, there is a significant paucity of research eliciting the relationship between the ToM and intelligence in typically developing children [20]. Therefore, we opted to study the relationship between the ToM and intelligence along with its various domains in children over broad IQ ranges and with out any psychiatric morbidity.

## Methods

### Setting and sample

We recruited participants from 12 mainstream schools and four special schools for children with Intellectual Disability (ID) in the Vellore educational district, Tamilnadu, Southern India. The schools represent the higher (Private ICSC board schools), middle (Private matriculation board schools); lower socio-economic (Public state board schools) backgrounds and they represent the literate late childhood population in India.

### Sample size estimation

We calculated sample size to identify correlation (rho = 0.3) between ToM and IQ. Keeping alpha error, beta error and *a priori *power at 5%, 20%, and 80% respectively, the sample size needed was 84, for a two-tailed evaluation. As we anticipated 20% drop out between the two points of assessment, we recruited at least 100 children in this study.

### Selection criteria

We included children of 8 to 11 years of age with their caregivers if they were willing to participate in the study. We excluded children with psychiatric morbidity, long-term physical illness, neurological deficits or disabilities, special sensory deficits, severe behavioral problems, long-term use of any medication, children who had already received prior training for ToM tasks and those unwilling to provide verbal assent or informed consent to participate in this study.

### Materials

#### Theory of mind

**Figure 1:**
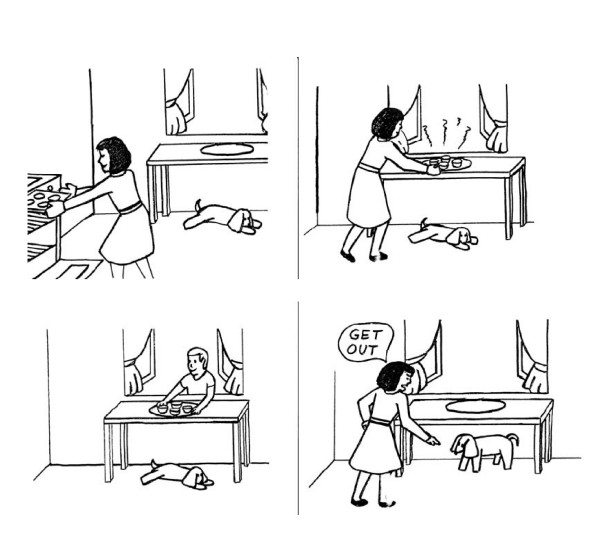
Sample pictures from the false belief domain in picture sequencing task.

The *Unexpected contents Theory of Mind Task *has a set of questions on the child's description of the appearance, reality, representational change and false belief variables with one point for every correct response [21]. We used this measure to screen the ability of the children to participate in further ToM assessment and to gain additional information on ToM. Detailed assessment of ToM was done using the *Picture Sequencing Task *(PST). The choice of non-verbal illustrative PST as the tool for ToM assessment alleviated the consequences of two major confounders, general language ability and working memory. PST measures the ToM ability by assessing false belief reasoning and general sequencing ability by employing 14 sequences, which included two practice, four false belief, four mechanical and four social script sequences. Each sequence consists of a series of four pictures made up of black and white sketches and some sample pictures are given in Figure 1. Averaging the mean scores of mechanical and social script sequences provided the General Sequencing Ability. The final score in PST is calculated by subtracting the general sequencing ability from the mean score of false belief sequences and therefore can be represented with a negative score [7]. The PST has also been used in children to study ToM by Langdon [22] who also provided us with the measure to be used in this study.

#### Intelligence

The *Binet-Kamat Scale of intelligence *[23] is the Indian adaptation of the 1934 version of Stanford-Binet Scale of Intelligence. Some of tests, items and materials were amended to suit Indian conditions, such as Indian coins, typically Indian pictorial scenes, vocabulary and Indian concepts. This intelligence scale assessed the child's skills in nine domains: language, meaningful memory, non-meaningful memory, conceptual thinking, verbal reasoning, non-verbal reasoning, numerical reasoning, visuomotor coordination and social intelligence.

#### Adaptive Behavior

The *Vineland Adaptive Behavior Scale *(VABS) assesses the social competence of children with or without disabilities from birth to 19 years of age under four domains and 11 sub-domains [24].

#### Psychopathology

The *Strength Difficulty Questionnaire *(SDQ) has 25 items that screen for psychological strengths and psychiatric disorders among children as well as categorize individuals with low needs; some needs and high needs [25]. It identifies children with conduct disorder, hyperactivity, depressive and anxiety disorders needing various interventions [26].

### Interview and assessment

APR, a qualified psychiatrist approached the children and their primary caregivers to enroll in this cross sectional study, according to the protocol approved by the hospital's Institutional Review Board. He obtained written informed consent from the primary care giver and verbal assent from the child before data collection. The interview and assessment protocol during the first day of evaluation required approximately two hours to complete. It consisted of three sections: (i) a face-to-face interview with open ended questions documenting the socio demographic data (ii) a structured assessment to screen for psychopathology using SDQ and for the ability to participate in ToM assessment using Unexpected Contents Theory of Mind task (iii) an in-depth ToM assessment with PST. During the second day of evaluation, SY, an experienced psychologist independently measured the intelligence and adaptive behavior using the BKT and the VABS respectively. Thus, we assessed 105 children of 8–11 years of age who satisfied the selection criteria and consented to participate in the study.

### Data analysis

Preliminary checks of skewness and kurtosis and the one sample Kolmogorov Smirnoff tests verified that our data were suitable for parametric analysis. Firstly, we analyzed the socio-demographic data, cognitive profile, adaptive behaviors and ToM details for all the participants using descriptive statistics. Secondly, we grouped the participants into three IQ-based groups based on the conventional nomenclature [27]: below average (IQ < 90); average (IQ 90–110); and above average (IQ > 110) intelligence. We compared the groups using Chi-square tests for categorical variables and one-way ANOVA for continuous variables. Thirdly, we analyzed the linear correlation between the ToM and various domains of general intelligence using Pearson's correlation coefficient tests. Finally, we also conducted the multiple linear regression analysis with ToM as the dependant variable to account for the effects of possible confounders. All tests used two-tailed analysis and a P value of less than 0.05 was considered significant. We analyzed our data using the statistical software package, SPSS 16.0.

## Results

Ninety-five children completed the study. The overall dropout rate was 9.52% and the reasons for attrition included withdrawal of consent by parents (N = 4), uninformed change of residence (N = 4) as well as on new information regarding past medical history (N = 2). We stratified those 95 children in to three groups of sub average (N = 33), average (N = 31) and above average (N = 31) intelligence. These groups when compared significantly differed on six socio demographic variables namely, chronological age, type of school, monthly income of the family, father's education, mother's education and mother's age. We considered these variables as potential confounders for our analyses. The socio-demographic, participant and family characteristics of completers are presented in Table 1.

**Table 1:** Socio demographic profile of children and their families (N = 95)

**Variable**	**Total** **N (%)**	**Below average**	**Average**	**Above average**	**χ^2^/F, df**	**P value** **(P*rep*)**
Gender:						
Male	42 (44)	15	17	10	2.87, 2	0.2
Female	53 (56)	18	14	21		(0.82)
Chronological age (in years):						
Mean (SD)	10.19 (0.86)	9.85 (0.81)	10.71 (0.58)	10.09 (0.73)	10.39, 2	**0.001** **(0.99)**
Type of school:						
Private	60 (63)	9	20	31	36.37, 2	**0.001**
Government	35 (37)	24	11	0		**(0.99)**
Type of Family:						
Nuclear	79 (83)	30	24	25	2.34, 2	0.3
Joint	16 (17)	3	7	6		(0.77)
Father's education						
No formal	6 (6)	6	0	0		
education						
School	42 (44)	23	14	5	38.56, 4	**0.001**
College	47 (50)	4	17	26		**(0.99)**
Mother's education						
No formal	6 (6)	5	1	0		
education						
School	46 (48)	25	15	6	35.19, 4	**0.001**
College	43 (45)	3	15	25		**(0.99)**
Age of father (in years)						
Mean (SD)	40.92 (6.30)	40.45 (8.56)	40.35 (4.60)	42.42 (5.62)	1.41, 2	0.2 (0.82)
Age of mother (in years)						
Mean (SD)	35.08 (5.40)	32.64 (6.06)	35.61 (4.86)	37.16 (4.17)	6.51, 2	**0.002 (0.99)**
Number of siblings:						
None	14 (15)	3	4	7	2.44, 2	0.2
One or more	81 (85)	30	27	24		(0.82)
Birth order:						
First	59 (62)	20	21	18		
Middle	7 (7)	5	2	0	7.46, 4	0.1
Last	29 (31)	8	8	13		(0.88)
Monthly family Income (Indian rupees/month)	13651 (18916)	2874 (4786)	12048 (17598)	26725 (21909)	17.35, 2	**0.002** **(0.99)**
F/H of psychiatric illness:						
Present	9 (10)	5	3	1	2.65, 2	0.2
Absent	86 (90)	28	28	30		(0.82)

F/H: Family History

Psychopathology assessment demonstrated low needs on hyper activity, conduct problems, emotional symptoms, peer problems and total difficulties domains. Participants also scored favorably on pro social domain with mean (sd) of 9.14(0.86) and thus we ruled out any major psychiatric morbidity among them. In addition, the principal investigator, a qualified psychiatrist, ruled out any ICD-10 [28] based psychiatric morbidity with face-to-face clinical interviews. We present the psychological characteristics of the participants in Table 2.

**Table 2:** Psychological profile of children who participated in this study (N = 95)

**MEASURE**	**DOMAIN**	**MEAN (SD)**
Picture Sequencing Task	Social script	4.16 (1.73)
	Mechanical	3.98 (1.67)
	False belief	3.25 (1.49)
	General Sequencing Ability	4.07 (1.59)
	**Theory of Mind ability**	**- 0.82 (1.29)**
Binet Kamat Test	Mental Age (in years)	9.53 (3.12)
	Language (in years)	10.01 (3.52)
	Meaningful memory (in years)	9.23 (2.66)
	Non meaningful memory (in years)	8.34 (2.72)
	Conceptual thinking (in years)	10.99 (2.24)
	Non verbal thinking (in years)	8.89 (4.36)
	Verbal reasoning (in years)	12.32 (1.09)
	Non verbal reasoning (in years)	9.25 (2.98)
	Visuo motor (in years)	8.90 (1.87)
	Social intelligence (in years)	9.76 (2.91)
	**Intelligence Quotient (IQ)**	**92.76 (29.04)**
Vineland Adaptive Behavior Scale	Adaptive Behaviour composite age equivalent (in years)	8.73 (3.12)

The mechanical (rho = 0.74, P = 0.001), social script (rho = 0.65, P = 0.001), general sequencing ability (rho = 0.77, P = 0.001) and false belief scores (rho = 0.70, P = 0.001) of PST were significantly correlated with IQ. The selective accuracy Theory of Mind score was calculated by subtracting the general sequencing ability scores from the mean score of false belief sequences.

The linear correlation between ToM and IQ was not significant (rho = -0.14, P = 0.17; P*rep *= 0.83). Among the sub domains of intelligence, only visuomotor (rho = -0.26, P = 0.02) and social intelligence (rho = -0.29, P = 0.003) were significantly related to ToM. We present the bivariate Pearson correlation matrix between ToM and various domains of intelligence in Table 3. The total score of Unexpected Contents Theory of Mind task did not correlate with the Intelligence Quotient (rho = -0.15; P = 0.14). ToM did not have significant relationship with IQ within below average (rho = -0.26, P = 0.2; P *rep *= 0.82), average (rho = -0.27, P = 0.2; P *rep *= 0.82) and above average (rho = 0.05, P = 0.8; P *rep *= 0. 57) intelligence groups. Though, ToM significantly differed between the three intelligence groups (F = 7.86, df = 2; P = 0.001), children with above average intelligence [mean (sd) = -0.35(0.79)] had better ToM than those with below average intelligence [mean (sd) = -0.61(1.51)] who in turn had better ToM than those with average intelligence [mean (sd) = -1.50 (1.17)].

**Table 3:** Correlation^a ^matrix between Theory of Mind ability and intelligence domains of participants

	**ToM**	**L**	**MM**	**NM**	**C T**	**NVT**	**VR**	**NR**	**VM**	**SI**	**IQ**
**ToM**	1	0.16 (0.13) [0.86]	0.20 (0.06) [0.91]	0.14 (0.20) [0.82]	0.21 (0.08) [0.89]	-0.25 (0.07) [0.90]	0.10 (0.54) [0.67]	-0.09 (0.40) [0.72]	**-0.26 (0.02)** **[0.95]**	**-0.30** **(0.003)** **[0.98]**	-0.14 (0.17) [0.83]
**L**		1	0.89**	0.81**	0.71**	0. 90**	0.33*	0.81**	0.81**	0.91**	0.93**
**MM**			1	0.80**	0.53**	0.85**	0.38*	0.77**	0.80**	0.85**	0.88**
**NM**				1	0.56**	0.78**	0.74**	0.79**	0.66**	0.77**	0.86**
**C T**					1	0.57**	*0.26 (0.11)*	0.62**	0.51**	0.68**	0.80**
**NVT**						1	-*0.39* *(0.07*)	0.85**	0.83**	0.90**	0.90**
**VR**							1	*0.29 (0.09)*	*0.10* *(0.59)*	*0.09* *(0.57)*	0.48*
**NR**								1	0.62**	0.75**	0.84**
**VM**									1	0.81*	0.79**
**SI**										1	0.92**
**IQ**											1

ToM = Theory of Mind ability; L = Language; MM = Meaningful memory; NM = Non meaningful memory; CT = Conceptual thinking; NVT = Non verbal thinking; VR = Verbal reasoning; NR = Nonverbal reasoning; VM = Visuo motor; SI = Social Intelligence; IQ = Intelligence Quotient.

^a ^Two tailed Pearson correlation coefficient (P value) [P *rep *Value]

*Significance at the level of P = 0.05; ** Significance at the level of P = 0.001

The multiple linear regression analysis with ToM as the dependent variable also demonstrated the lack of a significant relationship between ToM and IQ when the above stated six socio-demographic confounders were controlled [β(SE) = -0.24 (0.01), t = -1.59, P = 0.12; P*rep *= 0.86]. The social intelligence domain [β(SE) = -0.55 (0.06), t = -3.75, P = 0.001; P*rep *= 0.99] and the visuomotor domain [β(SE) = -0.32 (0.09), t = -2.43, P = 0.02; P*rep *= 0.95] continued to exhibit significant relationship with ToM. When we further controlled for the effects of VABS adaptive behavior composite age equivalent, the lack of a significant relationship between ToM and IQ [β(SE) = 0.02 (0.01), t = 0.05, P = 0.96; P*rep *= 0.51] and significant relationship of the social intelligence domain [β(SE) = -0.91 (0.11), t = -3.75, P = 0.001; P*rep *= 0.99] and the visuomotor domain [β(SE) = -0.49 (0.11), t = -2.57, P = 0.012; P*rep *= 0.96] remained. Other domains of intelligence did not have significant relationship with ToM.

## Discussion

Our findings suggest that Theory of Mind is a distinct cognitive ability and not related to either general intelligence or its domains after controlling the confounders. ToM was related to social intelligence in our study and has also been documented before [29]. This relationship between the ToM and social intelligence may not only reflect the association between the manifest social intelligence in the form of adaptive behavior but a true link between the social intelligence and ToM. This hypothesis needs further testing.

Language has been previously considered as having a robust linear relationship with the ToM even after accounting for the children's age and the verbal complexity of the tasks employed [30]. However, the role of the culture and language as inherent components of the measures used to assess ToM has not been controlled in the past studies [31,32]. In our study when we applied a non-verbal mode of ToM assessment and thus controlling the role of language and verbal skills inherent to the ToM measure, we demonstrated that ToM's correlation with language ability was not significant. The verbal and non-verbal memory functions were not related to ToM in our study and this finding has been noted previously [15]. The significant relationship noted between the visuomotor ability and ToM has not been documented in the past and in our study this relationship could be attributed to the nature of the measure, because PST involves general sequencing ability based on the visuomotor skills.

The main limitations of the study are, the cross-sectional nature of the study and selecting children within particular age range that possibly reduce the stability of our findings in children whose cognitive domains are continuously developing. However, the strengths of our study are firstly, studying children from sub average to above average intelligence whereas most of the previous studies have used children with intellectual disability [16]. Secondly, earlier studies did not control for the confounding effect of psychopathology on the relationship between intelligence and ToM [18,20], and we have ruled out any psychopathology that could confound this relationship. Thirdly, data collection by two independent masked investigators and a fair follow up of the prerequisite sample size reduced the observer and attrition bias respectively. Then, the baseline differences between different intelligence groups were controlled statistically to remove their confounding effects on the relationship between IQ and ToM. Finally, the selection of participants from clinic and community population strengthens the generalizability of the findings.

## Conclusion

Our study demonstrates a lack of significant linear relationship of ToM with the intelligence domains, language and memory as well as supports the argument that ToM exists as a specific independent cognitive domain. This study clarifies a puzzle in ToM research, facilitates interpretation of previous data and provides the impetus for pursuing neuro-cognitive studies to demarcate the biological systems underlying this cognitive domain. Our findings have potential implications in the fields of mental health, rehabilitation and education. Although it is standard research practice to interpret the study findings based on the null hypothesis and our study is robust in its results, replication of our findings will further establish this answer to the ToM-IQ riddle. Therefore, future studies should focus on the relationship between the intelligence and ToM with longitudinal designs and in cross-cultural settings.

## List of abbreviations

BKT: Binet Kamat Scale of intelligence; ID: Intellectual Disability; IQ: Intelligence quotient; SDQ: Strength Difficulty Questionnaire; ToM: Mind reading or Theory of Mind; VABS: Vineland Adaptive Behavior Scale.

## Competing interests

The authors declare that they have no competing interests.

## Authors' contributions

APR participated in designing this study, collected the data, performed statistical analyses and drafted the manuscript. SY collected the data and helped to draft the manuscript. ALR helped in data collection. PSSR conceived this study, designed and coordinated it, helped in the data analysis and corrected the final version of this manuscript.
